# Prevalence and trends of selected urologic conditions for VA healthcare users

**DOI:** 10.1186/1471-2490-6-30

**Published:** 2006-11-03

**Authors:** Min-Woong Sohn, Huiyuan Zhang, Brent C Taylor, Michael J Fischer, Elizabeth M Yano, Christopher Saigal, Timothy J Wilt, the Urologic Diseases in America Project

**Affiliations:** 1 Midwest Center for Health Services and Policy Research, Edward Hines, Jr. VA Hospital, Hines, IL, USA; 2 Feinberg School of Medicine, Northwestern University, Chicago, IL, USA; 3 Minneapolis VA Center for Chronic Disease Outcomes Research, Minneapolis, MN, USA; 4 University of Illinois Medical Center/VAMC, Chicago, IL, USA; 5 Center for the Study of Healthcare Provider Behavior, Sepulveda, CA, USA; 6 RAND/UCLA, Los Angeles, CA, USA; 7 University of Minnesota School of Medicine, Minneapolis, MN, USA

## Abstract

**Background:**

Conducted as part of the Urologic Diseases in America project whose aim was to quantify the burden of urologic diseases on the American public, this study focuses on Veterans Health Administration (VHA) users as a special population to supplement data on overall prevalence rates and trends in the United States. Veterans comprise 25% of the male population 18 years or older and contribute substantially to the overall burden of urologic conditions. The objective of this study is to describe the prevalence rates and trends of urologic cancers and selected benign conditions from 1999 to 2002 for VHA users.

**Methods:**

VHA administrative files for 1999 – 2002 and Medicare claims files for the same years were used to identify those who had a diagnosis of qualifying urologic conditions.

**Results:**

Among the conditions evaluated, prostate cancer was listed as a primary diagnosis for 5.4% of VHA users in 2002, followed in decreasing prevalence by erectile dysfunction (2.9%), renal mass (1.5%), interstitial cystitis (1.4%), and prostatitis (1.1%). Age-adjusted rates showed significant increases for renal mass (31%), interstitial cystitis (14%), and erectile dysfunction (8%) between 1999 and 2002. Systematic variations in prevalence rates and trends were observed by age, race/ethnicity, and region. Those in the Western region generally had lower age-adjusted prevalence rates and their increases were also slower than other regions. Addition of Medicare data resulted in large increases (21 to 489%) in prevalence among VHA users, suggesting substantial amount of non-VA urological care provided to VHA users.

**Conclusion:**

Prevalence rates for many urologic diseases increased between 1999 and 2002, which were not entirely attributable to the aging of veterans. This changing urologic disease burden has substantial implications for access to urologic care and treatment capacity, especially in light of the level of urologic care delivered to veterans by Medicare providers outside the VA. Further study on the factors associated with these increases and how they affect the patterns, cost, and quality of care in veterans is needed.

## Background

Urologic diseases can result in considerable morbidity, mortality and healthcare resource utilization [1-5]. An accurate estimation of their prevalence is crucial in predicting future costs and demands for related services, understanding patterns of care and planning for resources needed to meet the demand.

The objective of this study is to describe the prevalence rates of urologic cancers and selected benign urologic conditions and their trends between 1999 and 2002 for those who have been using healthcare from the Veterans Health Administration (VHA). Conducted as part of the Urologic Diseases in America (UDA) project whose aim was to quantify the burden of urologic diseases on the American public [2-5], this study focuses on VHA users as a special population to supplement data on overall prevalence rates and trends in the United States. Veterans comprise 25% of the male population 18 years or older [6] and contribute substantially to the overall burden of urologic conditions. About 3.8 million veterans (14% of all veterans) used healthcare in the VHA in 2000. The overall prevalence of, and patterns of care for urologic conditions may be seriously underestimated without data from the VHA [7].

## Methods

### Data sources and population

The Institutional Review Board of the Edward Hines, Jr. VA Hospital approved the study. The VHA Medical SAS Inpatient and Outpatient Datasets [8] for 1999 – 2002 and all Medicare claims files for the same years were used to identify those who were diagnosed with qualifying urologic diseases by VHA or Medicare providers during the four years [9-12].

For each year, the study population consisted of all VHA users of age 18 years or older who were veterans and used either inpatient or outpatient care in the VHA in the current or previous year.

Because some of the urologic conditions may occur almost entirely among individuals of a certain age range or gender, the base population was specifically defined for each disease and only the appropriate base population was searched to identify individuals diagnosed with a disease. For erectile dysfunction, peyronie's disease, infertility, undescended testis, hypospadias, prostatitis, and testis cancer, the base population was all male VHA users. For prostate cancer, the base population was further limited to male users aged 40 or older, and for bladder cancer, all users aged 40 or older. For all other conditions, it was all users 18 years or older.

### Identification of urologic conditions

In this study, 13 urologic conditions selected for Phase 3 of the UDA project, excluding four perinatal conditions that are not applicable to the VHA user population, were examined [3]. Five other urologic conditions had been studied at earlier phases and their results published [4,5]. Both *International Classification of Diseases, 9*^*th *^*Version, Clinical Modification *(ICD-9-CM) diagnostic and procedure codes for inpatient records and ICD-9-CM diagnostic codes and *Common Procedure Terminology, 4*^*th *^*Version *(CPT-4) procedure codes for outpatient records were used to detect conditions. A complete list of diagnostic and procedure codes used to identify patients with qualifying conditions is shown in the Appendix (see Additional file 1).

### Demographic variables

Prevalence rates and trends were characterized by age, race/ethnicity, gender, non-VA insurance status, and region of the country. Age was computed using veterans' dates of birth as age on January 1^st ^of each year. Race/ethnicity data were obtained from the VHA files and, when missing, were supplemented by those in the Medicare Denominator files [13].

Non-VA insurance status was obtained from the VHA Outpatient Medical SAS Datasets and was determined in five categories, including those with no insurance (self-pay), Medicare, Medicaid, private insurance, and other insurance.

Region was coded separately for each year based on where the healthcare episodes occurred, not where the patient resided. However, if a veteran received healthcare from providers in two or more regions in the same year, region was assigned based on the patient's zipcode of residence. Patients were grouped into four regions, Northeast, Midwest, South, and West, as defined by the U.S. Census Bureau [14].

### Statistical analysis

Prevalence rates were calculated as the ratio of all persons in the base population to the persons having a qualifying condition listed as a *primary *diagnosis in a given year. These rates were all age-adjusted to the 2000 VHA population using direct standardization [15,16]. To examine disease trends, we computed percent change in prevalence rates for the eight most prevalent diseases using 1999 rates as the base and the trends were tested for statistical significance using Mantel-Haenszel chi-square test for trend [17]. All computation of prevalence rates and trends were conducted using SAS version 9.3.

## Results

### Overall prevalence rates and trends

Table 1 shows raw counts and age-adjusted prevalence rates per 100,000 VHA users of selected urologic conditions listed as *primary *diagnoses for years 1999 – 2002. The base population of all VHA users (18 years or older) was 3.7 million in 1999 increasing to 4.8 million in 2002. This represents a 28% increase over the four year period. All other base populations showed the same upward trends as the overall population. The average age of VHA users increased from 58.9 years in 1999 to 62.0 years in 2002. About 43% of VHA users were elderly (65 or older) in 1999, while 49% were in 2002.

Among the conditions studied, prostate cancer was the most common urologic disease in 2002 (5,434/100,000 in the base population), followed by erectile dysfunction (2,884/100,000), renal mass (1,584/100,000), interstitial cystitis (1,358/100,000), prostatitis (1,128/100,000), and bladder cancer (943/100,000).

Conditions such as erectile dysfunction and renal mass were detected mainly as secondary diagnoses (data not shown). While 60% of all renal mass cases were listed as a secondary diagnosis for all four years, increasingly higher percentage of erectile dysfunction cases were listed as a secondary diagnosis from 33% in 1999 to 54% in 2002. In contrast, only about 20% of all cancers considered in this study were listed as secondary diagnoses (data not shown).

Over the four-year period, renal mass (31%; chi-square test for trend, p < 0.01), interstitial cystitis (14%; p < 0.01), erectile dysfunction (8%; p = 0.03), kidney cancer (7%; p = 0.87), and prostate cancer (4%; p = 0.09) showed increases in age-adjusted rates, although not all were statistically significant. Prostatitis and urethral stricture decreased by 10% and 13% over the same period, respectively.

The changes in crude rates were even larger. Most notably, all three cancers increased 10% or more (data not shown). Raw counts increased much more rapidly than rates. Between 1999 and 2002, raw counts increased by 48% for renal mass, 38% for interstitial cystitis, 36% for prostate cancer, 35% for kidney cancer, 33% for bladder cancer, 30% for erectile dysfunction, 21% for urethral stricture, and 20% for prostatitis (chi-square tests for trend, p < 0.01 for all conditions).

### Rates and trends by selected demographic categories

Table 2 shows prevalence rates in 2002 and their four-year changes from 1999 to 2002 of the eight most prevalent urologic conditions by selected demographic categories. Rates for all variables other than age are age-adjusted using 2000 as the reference year.

Age-specific rates indicate that the prevalence generally increased with age for all conditions. The largest increases in prevalence rates occurred among those who were 75 or older for five of the eight conditions. In contrast, prostatitis and urethral stricture were decreasing for all but one age group (85 +). The diagnosis of erectile dysfunction increased for all but one age group and the largest relative increase was observed for those younger then 45 years of age.

Large gender differences in prevalence among VHA users were found for bladder cancer, kidney cancers, renal mass, and urethral stricture; all of them were more than 3 times more prevalent among male than female users. Interstitial cystitis by contrast was almost twice as prevalent among female than male users.

There were considerable variations in prevalence across race/ethnicity groups. Of all groups, Whites had the highest prevalence in six of the eight conditions and Blacks had the highest in the other two conditions, erectile dysfunction and urethral stricture. The rates of bladder cancer and interstitial cystitis were respectively 2.4 and 1.6 times more prevalent among Whites than among the next most prevalent group, ''Others'' (1,222 vs. 507 per 100,000; p < 0.001) for bladder cancer, and Blacks for interstitial cystitis (1,735 vs. 1,103 per 100,000; p < 0.001). The rates for prostate cancer were over 6,000/100,000 for both Whites and Blacks, but those for Hispanic and Other groups were lower than 4,000/100,000. The rates of urethral stricture were considerably higher than 500/100,000 for Whites and Blacks but lower than 400/100,000 for Hispanics and Others.

VHA users who were also Medicare beneficiaries showed the highest prevalence rates for all of the eight conditions, consistent with the rates for those in 65 or older age groups. Those with Medicaid showed the most rapid increase in prevalence for prostate cancer (16%), kidney cancer (21%), renal mass (24%), and erectile dysfunction (18%). Those with no insurance or Medicaid coverage had the highest increases in prevalence of erectile dysfunction of all groups.

VHA users who obtained healthcare in the Northeast region had the highest prevalence rates for prostate cancer, bladder cancer, kidney cancer, interstitial cystitis, while those living in the West region consistently showed the lowest rates for six of the eight conditions. The West region also showed the lowest increases in prevalence in seven of eight conditions. For erectile dysfunction, the South region had the highest prevalence and showed the most rapid increase.

### Rates and trends by data source

Table 3 shows age-adjusted prevalence rates for qualifying conditions, computed based on the VHA data alone and the combined VHA and Center for Medicare and Medicaid Services (CMS) data for all VHA users. The percentage of additional cases identified by adding CMS data varied greatly by condition ranging from 21% (infertility in 2000) to 489% (posterior urethral valves in 2002).

**Figure 1:**
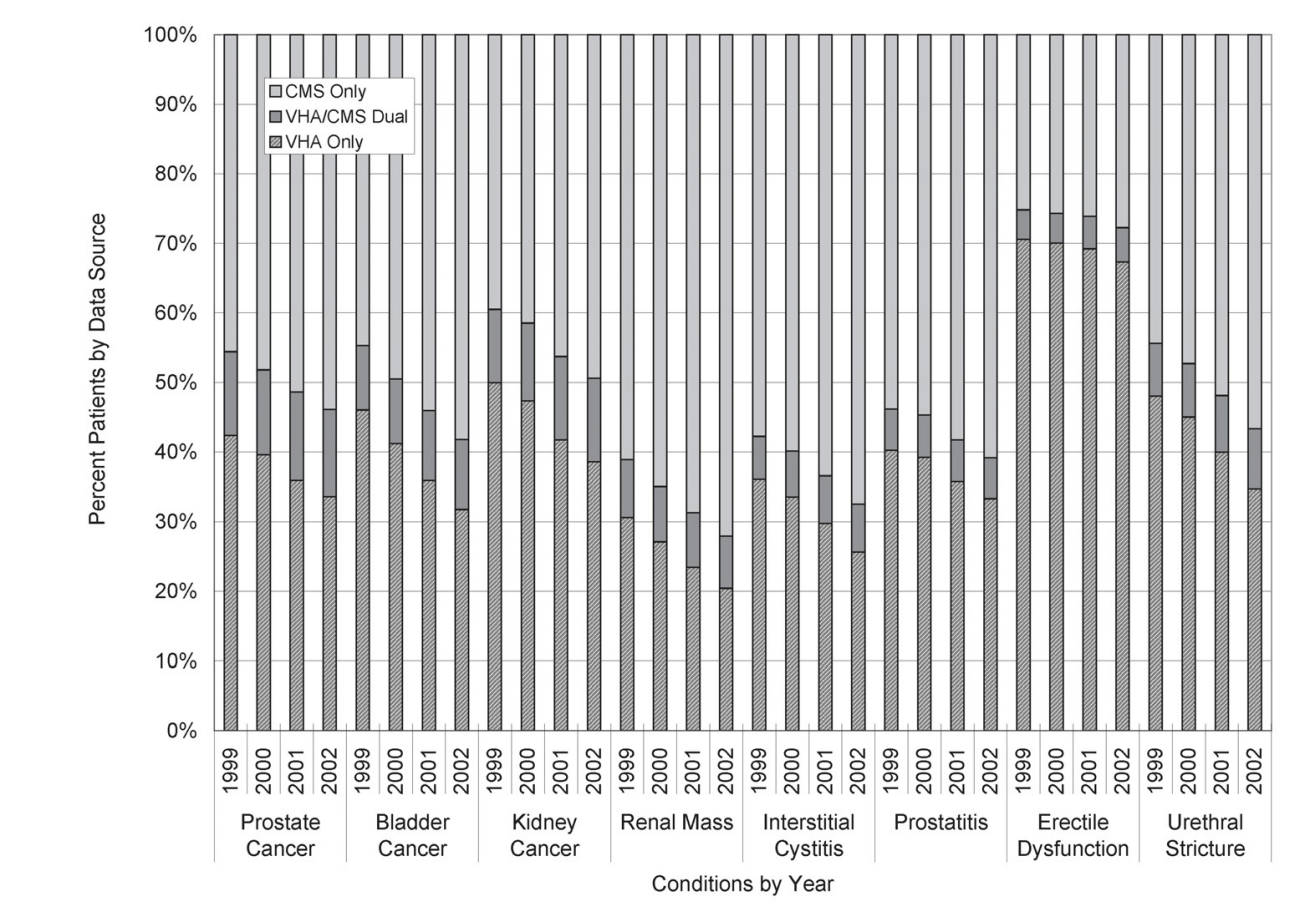
Percent of Patients Diagnosed with a Qualifying Condition by Data Source, 1999 – 2002

Figure 1 shows percent distribution of patients by year and source of data for the eight most prevalent conditions. Age-adjusted prevalence rates of patients diagnosed by Medicare providers alone increased significantly for five of the eight conditions (except kidney cancer, prostatitis, and urethral stricture), while the rates for all conditions diagnosed by both VHA and Medicare providers each year remained unchanged and those diagnosed in VHA hospitals alone actually decreased significantly for five conditions (except bladder cancer, renal mass, and erectile dysfunction). Thus, for example, there were only 4% increase in overall age-adjusted prevalence rates for prostate cancer between 1999 and 2002. The trends decomposed by data source however shows that the prevalence among VHA users decreased by 12%, while that among Medicare users increased by 20% (data not shown).

## Discussion

Our results show a high and increasing prevalence of several urologic conditions among VHA users. Among the conditions we examined, most commonly listed as a primary diagnosis for VHA users were prostate cancer (5.4%), erectile dysfunction (2.9%), renal mass (1.5%), interstitial cystitis (1.4%), and prostatitis (1.1%) in 2002.

Many urologic conditions were diagnosed increasingly more over the four-year study period. Erectile dysfunction and renal mass showed the largest increases. In particular, these two were the conditions commonly listed as secondary diagnoses, suggesting that these conditions were likely diagnosed, evaluated and treated in non-urologic settings in the context of other co-morbid conditions.

The increase in the prevalence of erectile dysfunction coincides with the approval and availability of effective oral therapies such as, sildenafil, in 1998 [18]. The VHA started to cover sildenafil as a treatment option for erectile dysfunction in 2001 [19,20]. Thus, the anticipation and availability of this drug might have caused VHA users to seek care for erectile dysfunction as a secondary diagnosis when they see primary care providers.

The more than four-fold greater increase over time in the prevalence of renal mass compared to kidney cancer (31% versus 7%) may be attributed to more frequent use of imaging studies (CT scans, ultrasounds, and MRI) for non-urologic conditions. Future research is needed to evaluate the increased health care costs and complications associated with additional diagnostic evaluations versus potential benefits of earlier and more frequent use of kidney cancer screening and consequent identification of renal masses.

While a diagnosis of prostatitis and urethral stricture declined, prostate, bladder, and kidney cancers all showed increasing trends in counts and crude rates over this period. However, these trends appear to be mostly attributable to the aging of veterans. When the prevalence rates were age-adjusted, the four-year trends were not statistically significant for any of these cancers.

Significant age, geographic, and racial variations in these conditions were also observed. Variations by age groups were consistent with expectation for all most prevalent conditions. Another interesting finding was the consistent pattern by which the VHA users in the Western region had the lowest prevalence rates and smallest increases in many of the urologic conditions. We do not know whether this pattern is attributable to demographic composition of the study population, to different practice patterns or the way services are organized and provided across regions. These patterns are not adjusted for other demographic factors and co-morbidities and should be interpreted cautiously. Further study is needed to understand the geographic variations in the prevalence rates and their changes over time of these urologic conditions.

Another key finding was the increasing proportions of VHA users turning to Medicare hospitals for the diagnosis of some urologic conditions. These trends were significant for five of the eight most prevalent conditions even after the rates were age-adjusted, suggesting that these trends could not be explained by the aging of veterans. For example, the VHA cared for 12% less prostate cancer patients, while Medicare providers cared for 20% more, in 2002 than in 1999. A number of factors such as access to care and financial concerns may have contributed to these trends. Since these findings have important implications in the resource use and management of these patients for both the VHA and the CMS, the impact on disease prevalence and/or patterns of care for these patients warrants further research.

As with any administrative database, limitations exist in regard to the accuracy and completeness of the coded information. Because VHA providers are not reimbursed for a diagnosis or a treatment, less incentive may exist for them to fully code all conditions in contrast to Medicare providers. Thus, our findings likely are conservative estimates of disease prevalence and may be one explanation why inclusion of Medicare data substantially increased overall prevalence rates.

## Conclusion

In conclusion, prevalence rates of several high priority urologic diseases including prostate, bladder, kidney cancers, renal mass and erectile dysfunction are increasing in veterans and show regional and racial variations. The comparison of the VHA and the Medicare data shows different trends in prevalence of several urologic conditions. Prevalence rates among veterans might be substantially underestimated without using other administrative claims data, including Medicare in combination with VHA data.

## Competing interests

The author(s) declare that they have no competing interests.

## Authors' contributions

MWS participated in the design of the study, oversaw statistical analyses of data, and drafted the manuscript.

HZ was responsible for data collection, management, statistical analysis, and edited the manuscript.

BCT participated in the design of the study, data analysis, and edited the manuscript.

MJF participated in the interpretation of results, and edited the manuscript.

EY participated in the design of the study, the interpretation of results, and edited the manuscript.

CS participated in the design of the study, the interpretation of results, and edited the manuscript.

TJW oversaw the 2^nd ^grant, participated in the design of the study, the interpretation of results, and edited the manuscript.

All authors read and approved the final manuscript.

## Pre-publication history

The pre-publication history for this paper can be accessed here:



## Supplementary Material

Additional file 1 Appendix: List of Urologic Conditions, their Base Populations, and Methods of Identification. The table is an appendix and shows the list of urologic conditions, their base populations, and methods of identification of conditions considered in the study. Click here for file

## Figures and Tables

**Table 1:** Counts and Age-Adjusted Prevalence Rates of Urologic Conditions Listed As Primary Diagnosis, 1999 – 2002*

Conditions	1999			2000			2001			2002		
	
	Count	Age	Adjusted Rate (95% CI)	Count	Age	Adjusted Rate (95% CI)	Count	Age	Adjusted Rate (95% CI)	Count	Age	Adjusted Rate (95% CI)
Male reproductive health												
Erectile Dysfunction	93,837	2,669	(2,652 – 2,685)	105,481	2,790	(2,773 – 2,807)	118,375	2,805	(2,788 – 2,822)	133,100	2,884	(2,867 – 2,901)
Peyronie's Disease	1,399	40	(38 – 42)	1,501	40	(38 – 42)	1,482	35	(33 – 37)	1,548	33	(31 – 35)
Infertility	2,751	75	(72 – 78)	2,822	75	(72 – 77)	3,051	76	(73 – 79)	3,146	74	(72 – 77)
Undescended Testis	235	7	(6 – 7)	252	7	(6 – 7)	286	7	(6 – 8)	227	5	(4 – 6)
Urethral diseases												
Hypospadias	269	8	(7 – 9)	263	7	(6 – 8)	291	7	(6 – 8)	308	7	(6 – 7)
Stricture	15,570	439	(432 – 445)	16,303	412	(406 – 418)	17,768	387	(381 – 393)	19,751	383	(377 – 389)
Interstitial Cystitis	42,638	1,195	(1,184 – 1,206)	49,710	1,256	(1,245 – 1,267)	61,500	1,344	(1,332 – 1,355)	69,469	1,358	(1,347 – 1,370)
Prostatitis	43,257	1,254	(1,243 – 1,266)	45,362	1,200	(1,189 – 1,211)	52,005	1,204	(1,193 – 1,215)	53,877	1,128	(1,118 – 1,139)
Prostate cancer	159,800	5,226	(5,202 – 5,250)	180,633	5,232	(5,208 – 5,256)	217,997	5,373	(5,349 – 5,398)	247,914	5,434	(5,409 – 5,459)
Bladder Cancer	29,725	938	(928 – 948)	33,016	926	(916 – 936)	39,345	942	(932 – 952)	44,397	943	(933 – 953)
Kidney Cancer	7,777	217	(213 – 222)	8,692	220	(215 – 224)	10,345	227	(222 – 231)	11,878	233	(229 – 238)
Renal Mass	42,725	1,209	(1,198 – 1,220)	52,117	1,317	(1,305 – 1,328)	66,790	1,444	(1,432 – 1,455)	82,287	1,584	(1,571 – 1,596)
Testis cancer	1,848	51	(49 – 53)	1,876	50	(47 – 52)	2,019	50	(48 – 52)	2,011	47	(44 – 49)

* Rates are per 100,000 persons in the base population

**Table 2:** Prevalence Rates and their Changes of 8 Urologic Conditions from 1999 to 2002, Primary Diagnosis by Selected Demographic Categories

		Prostate Cancer			Bladder Cancer			Kidney Cancer			Renal Mass		
		
		2002 Rate		Change	2002 Rate		Change	2002 Rate		Change	2002 Rate		Change
**Total**		5,434	(5,409 – 5,459)	4.0	943	(933 – 953)	0.5	233	(229 – 238)	7.4	1,584	(1,571 – 1,596)	31.0

**Age**													
	< 25							3	(-2 – 8)	-62.9	21	(6 – 35)	-50.5
	25 – 34							11	(6 – 16)	10.6	76	(64 – 89)	-16.2
	35 – 44	94	(81 – 107)	-26.6	50	(41 – 59)	-37.3	35	(29 – 41)	6.0	171	(158 – 184)	-2.3
	45 – 54	605	(589 – 621)	11.7	158	(150 – 166)	-15.3	98	(92 – 105)	0.3	357	(345 – 369)	3.5
	55 – 64	2,280	(2,248 – 2,312)	-7.8	443	(429 – 457)	-18.7	192	(183 – 201)	-1.8	722	(704 – 739)	2.9
	65 – 74	8,038	(7,987 – 8,089)	3.1	1,314	(1,294 – 1,335)	-0.9	375	(364 – 386)	5.1	2,478	(2,450 – 2,506)	30.2
	75 – 84	11,614	(11,547 – 11,681)	5.9	2,042	(2,014 – 2,070)	7.7	419	(406 – 432)	17.8	3,692	(3,655 – 3,729)	43.0
	85 +	11,336	(11,127 – 11,545)	18.0	2,134	(2,045 – 2,224)	21.4	296	(263 – 329)	6.9	4,075	(3,951 – 4,198)	52.6

**Gender**													
	Male	5,434	(5,409 – 5,459)	4.0	965	(954 – 975)	0.5	241	(236 – 246)	7.4	1,635	(1,622 – 1,648)	30.7
	Female				243	(214 – 272)	-3.2	71	(58 – 83)	10.6	462	(430 – 493)	53.4

**Race-Ethnicity**													
	White	6,318	(6,286 – 6,349)	3.7	1,222	(1,209 – 1,236)	-0.6	297	(290 – 303)	8.9	2,019	(2,001 – 2,036)	33.9
	Black	6,435	(6,360 – 6,511)	6.3	434	(414 – 453)	-7.2	217	(204 – 230)	7.5	1,604	(1,570 – 1,639)	20.4
	Hispanic	3,626	(3,498 – 3,753)	1.3	370	(330 – 410)	0.6	188	(161 – 215)	17.4	814	(758 – 870)	28.5
	Other	4,163	(3,973 – 4,352)	8.1	507	(442 – 573)	-8.8	214	(173 – 255)	-4.3	1,431	(1,325 – 1,537)	26.1

**Insurance Status**													
	No ins/self-pay	4,145	(4,118 – 4,172)	2.7	708	(697 – 719)	-0.9	186	(181 – 191)	5.7	1,105	(1,092 – 1,117)	22.7
	Medicare	9,518	(9,444 – 9,592)	-1.2	1,709	(1,678 – 1,739)	-3.6	409	(394 – 424)	6.4	3,238	(3,196 – 3,280)	24.0
	Medicaid	2,591	(2,061 – 3,121)	16.0	509	(280 – 739)	-16.2	175	(47 – 302)	20.6	1,014	(706 – 1,322)	24.3
	Private Ins/HMO	5,049	(4,989 – 5,109)	-5.6	803	(780 – 826)	-19.3	209	(197 – 221)	-10.2	1,417	(1,387 – 1,447)	13.9
	Other Insurance	2,261	(2,063 – 2,460)	3.0	379	(300 – 458)	-19.4	116	(75 – 158)	-36.5	642	(544 – 740)	-0.4

**Region**													
	Northeast	6,508	(6,440 – 6,577)	3.4	1,222	(1,192 – 1,251)	3.6	275	(262 – 289)	4.4	1,682	(1,649 – 1,715)	37.7
	Midwest	5,398	(5,339 – 5,457)	4.9	986	(962 – 1,011)	1.1	236	(225 – 248)	4.3	1,830	(1,798 – 1,862)	38.6
	South	5,467	(5,427 – 5,506)	3.8	903	(887 – 918)	2.4	237	(230 – 245)	8.6	1,676	(1,656 – 1,696)	28.7
	West	4,666	(4,624 – 4,709)	-0.1	776	(759 – 793)	-10.4	195	(187 – 203)	4.1	1,139	(1,119 – 1,158)	12.3

		Interstitial Cystitis			Prostatitis			Erectile Dysfunction			Urethral Stricture		
		
		2002 Rate		Change	2002 Rate		Change	2002 Rate		Change	2002 Rate		Change

**Total**		1,358	(1,347 – 1,370)	13.7	1,128	(1,118 – 1,139)	-10.0	2,884	(2,867 – 2,901)	8.1	383	(377 – 389)	-12.7

**Age**													
	< 25	439	(373 – 505)	8.1	144	(100 – 187)	-5.0	270	(211 – 330)	34.1	21	(6 – 35)	-57.6
	25 – 34	490	(458 – 522)	0.6	256	(230 – 281)	-17.1	662	(621 – 703)	45.7	62	(51 – 74)	-10.5
	35 – 44	503	(481 – 526)	1.6	394	(372 – 415)	-17.3	1,506	(1,465 – 1,548)	32.2	83	(74 – 92)	-21.4
	45 – 54	410	(397 – 423)	-3.5	571	(555 – 586)	-14.9	2,983	(2,947 – 3,018)	19.1	129	(122 – 136)	-18.9
	55 – 64	559	(543 – 575)	-15.5	787	(768 – 806)	-18.6	3,585	(3,545 – 3,625)	-1.9	211	(202 – 221)	-26.0
	65 – 74	1,934	(1,909 – 1,959)	15.5	1,833	(1,808 – 1,857)	-6.7	3,906	(3,870 – 3,941)	5.2	547	(534 – 560)	-13.9
	75 – 84	3,028	(2,994 – 3,062)	22.6	1,720	(1,694 – 1,745)	-8.5	2,204	(2,175 – 2,234)	6.2	839	(821 – 856)	-6.9
	85 +	3,349	(3,237 – 3,461)	37.8	1,305	(1,234 – 1,376)	1.2	926	(866 – 985)	19.8	1,047	(984 – 1,109)	0.1

**Gender**													
	Male	1,311	(1,300 – 1,323)	14.1	1,128	(1,118 – 1,139)	-10.0	2,884	(2,867 – 2,901)	8.1	396	(390 – 403)	-12.5
	Female	2,205	(2,136 – 2,274)	-2.4							98	(83 – 113)	-25.2

**Race-Ethnicity**													
	White	1,735	(1,719 – 1,751)	13.9	1,383	(1,368 – 1,397)	-9.7	3,059	(3,037 – 3,080)	7.1	474	(466 – 482)	-9.8
	Black	1,103	(1,074 – 1,131)	11.8	1,002	(974 – 1,030)	-7.9	4,618	(4,557 – 4,678)	15.3	428	(410 – 445)	-19.9
	Hispanic	729	(676 – 782)	-4.8	1,101	(1,035 – 1,167)	-17.8	3,212	(3,099 – 3,324)	19.5	311	(277 – 346)	-11.3
	Other	1,018	(928 – 1,107)	5.7	999	(909 – 1,089)	-7.7	2,647	(2,501 – 2,794)	4.7	284	(237 – 332)	-17.5

**Insurance Status**													
	No ins/self-pay	1,017	(1,005 – 1,029)	9.0	918	(906 – 929)	-14.0	2,709	(2,689 – 2,730)	9.6	290	(283 – 296)	-15.3
	Medicare	2,539	(2,502 – 2,577)	6.8	1,838	(1,806 – 1,870)	-12.3	3,280	(3,237 – 3,323)	3.9	714	(694 – 734)	-13.6
	Medicaid	1,037	(726 – 1,348)	-7.7	793	(512 – 1,074)	-37.0	3,497	(2,907 – 4,087)	17.9	323	(150 – 497)	-28.7
	Private Ins/HMO	1,267	(1,238 – 1,295)	2.6	1,060	(1,033 – 1,087)	-15.1	2,994	(2,949 – 3,038)	-1.3	350	(335 – 365)	-26.7
	Other Insurance	633	(536 – 730)	-0.2	768	(656 – 880)	-13.7	3,723	(3,477 – 3,969)	-4.2	224	(166 – 281)	-14.3

**Region**													
	Northeast	1,858	(1,824 – 1,893)	15.5	996	(970 – 1,022)	-7.7	2,471	(2,430 – 2,511)	6.5	397	(381 – 413)	-7.0
	Midwest	1,371	(1,344 – 1,399)	19.1	976	(952 – 1,000)	-3.3	2,841	(2,800 – 2,882)	4.5	400	(385 – 415)	-10.7
	South	1,334	(1,316 – 1,352)	12.2	1348	(1,329 – 1,366)	-12.2	3,118	(3,090 – 3,147)	14.6	402	(392 – 412)	-16.5
	West	1,030	(1,011 – 1,049)	0.7	980	(962 – 999)	-13.0	2,825	(2,793 – 2,857)	2.8	329	(319 – 340)	-14.2

* Rates per 100,000 persons in the base population. % change represents change in age-adjusted prevalence rates over 1999 – 2002.

**Table 3:** Comparison of Age-Adjusted Prevalence Rates per 100,000 in the Base Population Based on VHA Data and VHA/CMS Data*

	**1999 Rates (95% CI)**		
	**VHA**	**VHA/CMS**	**% Change**

Male reproductive health			
Erectile Dysfunction	1,996 (1,982 – 2,011)	2,669 (2,652 – 2,685)	34
Peyronie's Disease	27 (26 – 29)	40 (38 – 42)	48
Infertility	62 (59 – 64)	75 (72 – 78)	21
Undescended Testis	4 (4 – 5)	7 (6 – 7)	75
Urethral diseases			
Hypospadias	5 (4 – 5)	8 (7 – 9)	60
Stricture	244 (239 – 249)	439 (432 – 445)	80
Interstitial Cystitis	504 (497 – 511)	1,195 (1,184 – 1,206)	137
Prostatitis	579 (571 – 586)	1,254 (1,243 – 1,266)	117
Prostate cancer	2,843 (2,825 – 2,861)	5,226 (5,202 – 5,250)	84
Bladder Cancer	519 (511 – 526)	938 (928 – 948)	81
Kidney Cancer	131 (128 – 135)	217 (213 – 222)	66
Renal Mass	470 (463 – 477)	1,209 (1,198 – 1,220)	157
Testis cancer	40 (38 – 42)	51 (49 – 53)	28

			
	**2000 Rates (95% CI)**		

	**VHA**	**VHA/CMS**	**% Change**

Male reproductive health			
Erectile Dysfunction	2,073 (2,059 – 2,088)	2,790 (2,773 – 2,807)	35
Peyronie's Disease	26 (25 – 28)	40 (38 – 42)	54
Infertility	62 (59 – 64)	75 (72 – 77)	21
Undescended Testis	5 (4 – 5)	7 (6 – 7)	40
Urethral diseases			
Hypospadias	4 (3 – 5)	7 (6 – 8)	75
Stricture	217 (212 – 222)	412 (406 – 418)	90
Interstitial Cystitis	504 (497 – 511)	1,256 (1,245 – 1,267)	149
Prostatitis	543 (536 – 551)	1,200 (1,189 – 1,211)	121
Prostate cancer	2,709 (2,692 – 2,727)	5,232 (5,208 – 5,256)	93
Bladder Cancer	467 (460 – 475)	926 (916 – 936)	98
Kidney Cancer	128 (125 – 132)	220 (215 – 224)	72
Renal Mass	462 (455 – 468)	1,317 (1,305 – 1,328)	185
Testis cancer	38 (36 – 40)	50 (47 – 52)	32

			
	**2001 Rates (95% CI)**		

	**VHA**	**VHA/CMS**	**% Change**

Male reproductive health			
Erectile Dysfunction	2,071 (2,057 – 2,086)	2,805 (2,788 – 2,822)	35
Peyronie's Disease	22 (20 – 23)	35 (33 – 37)	59
Infertility	61 (58 – 63)	76 (73 – 79)	25
Undescended Testis	4 (3 – 5)	7 (6 – 8)	75
Urethral diseases			
Hypospadias	4 (3 – 5)	7 (6 – 8)	75
Stricture	186 (182 – 190)	387 (308 – 393)	108
Interstitial Cystitis	491 (484 – 498)	1,344 (1,332 – 1,355)	174
Prostatitis	502 (495 – 509)	1,204 (1,193 – 1,215)	140
Prostate cancer	2,611 (2,594 – 2,628)	5,373 (5,349 – 5,398)	106
Bladder Cancer	433 (426 – 440)	942 (932 – 952)	118
Kidney Cancer	122 (118 – 125)	227 (222 – 231)	86
Renal Mass	452 (445 – 458)	1,444 (1432 – 1,455)	219
Testis cancer	38 (36 – 40)	50 (48 – 52)	32

			
	**2002 Rates (95% CI)**		

	**VHA**	**VHA/CMS**	**% Change**

Male reproductive health			
Erectile Dysfunction	2,083 (2,069 – 2,098)	2,884 (2,867 – 2,901)	38
Peyronie's Disease	19 (18 – 21)	33 (31 – 35)	74
Infertility	59 (56 – 61)	74 (72 – 77)	25
Undescended Testis	3 (2 – 3)	5 (4 – 6)	67
Urethral diseases			
Hypospadias	4 (3 – 4)	7 (6 – 7)	75
Stricture	166 (162 – 170)	383 (377 – 389)	131
Interstitial Cystitis	441 (435 – 448)	1,358 (1,347 – 1,370)	208
Prostatitis	442 (435 – 448)	1,128 (1,118 – 1,139)	155
Prostate cancer	2,505 (2,488 – 2,521)	5,434 (5,409 – 5,459)	117
Bladder Cancer	394 (387 – 400)	943 (933 – 953)	139
Kidney Cancer	118 (115 – 121)	233 (229 – 238)	97
Renal Mass	442 (436 – 449)	1,584 (1,571 – 1,596)	258
Testis cancer	35 (33 – 37)	47 (44 – 49)	34

* VHA indicates prevalence rates based on VHA data alone; VHA/CMS, prevalence rates based on VHA and Medicare Data.

## References

[B1] Calhoun EA, McNaughton CM, Pontari MA, O'Leary M, Leiby BE, Landis RJ, Kusek JW, Litwin MS (2004). The economic impact of chronic prostatitis. Arch Intern Med.

[B2] Litwin MS, Saigal CS, Beerbohm EM (2005). The burden of urologic diseases in America. J Urol.

[B3] Litwin MS, Saigal CS, Yano EM, Avila C, Geschwind SA, Hanley JM, Joyce GF, Madison R, Pace J, Polich SM, Wang M (2005). Urologic diseases in America Project: analytical methods and principal findings. J Urol.

[B4] Pearle MS, Calhoun EA, Curhan GC (2005). Urologic diseases in America project: urolithiasis. J Urol.

[B5] Litwin MS, Saigal CS, Litwin MS and Saigal CS (2004). Urologic diseases in America: Interim Compendium.

[B6] Richardson C, Waldrop J (2005). Veterans: 2000.. http://www census gov/prod/2003pubs/c2kbr-22 pdf.

[B7] U.S. Department of Veterans Affairs (2000). Annual Performance Report FY 2000.

[B8] Murphy PA, Cowper DC, Seppala G, Stroupe KT, Hynes DM (2002). Veterans Health Administration inpatient and outpatient care data: an overview. Eff Clin Pract.

[B9] Veterans Affairs Information Resource Center (2003). VIReC Research User Guide: FY2002 VHA Medical SAS Inpatient Datasets.

[B10] Veterans Affairs Information Resource Center (2003). VIReC Research User Guide: FY2002 VHA Medical SAS Outpatient Datasets.

[B11] Veterans Affairs Information Resource Center (2003). Research findings from the VA Medicare data merge initiative: veterans' enrollment, access and use of Medicare and VA health services.

[B12] Hynes DM, Koelling K, Stroupe K, Arnold N, Sohn MW, al. Access and Use of Medicare and VA Services by Veterans. Med Care.

[B13] Sohn MW, Zhang H, Arnold N, Stroupe K, Taylor BC, Wilt TJ, Hynes DM (2006). Transition to the new race/ethnicity data collection standards in the Department of Veterans Affairs. Popul Health Metr.

[B14] U.S. Census Bureau (2006). Statistical Abstracts of the United States 2006, 125th Edition.

[B15] Klein RJ, Schoenborn CA (2001). Age adjustment using the 2000 projected U.S. population.

[B16] Anderson RN, Rosenberg HM (1998). Age standardization of death rates: implementation of the year 2000 standard.

[B17] Kuritz SJ, Landis JR, Koch GG (1988). A general overview of Mantel-Haenszel methods: applications and recent developments. Annu Rev Public Health.

[B18] Nathan PH (1999). A new era in the treatment of erectile dysfunction. Am J Cardiol.

[B19] Fink HA, Mac DR, Rutks IR, Nelson DB, Wilt TJ (2002). Sildenafil for male erectile dysfunction: a systematic review and meta-analysis. Arch Intern Med.

[B20] U.S. Department of Veterans Affairs Pharmacy Benefits Management-Medical Advisory Panel (1999). The primary care management of erectile dysfunction.

