# Supplementary material for: Prevalence and trends of selected urologic conditions for VA healthcare users
Source: BMC Urol. 2006 Nov 3;6:30. doi: 10.1186/1471-2490-6-30 (PMC1654168; doi:10.1186/1471-2490-6-30)
Supplement: Additional file 1 — Appendix: List of Urologic Conditions, their Base Populations, and Methods of Identification. The table is an appendix and shows the list of urologic conditions, their base populations, and methods of identification of conditions considered in the study. [file 1471-2490-6-30-S1.doc]

# Appendix

**List of Urologic Conditions, their Base Populations, and Methods of Identification**

| Condition | Base Population | Identified by |
| --- | --- | --- |
| Erectile dysfunction | All males | ICD-9 Dx: 302.71, 302.72, 302.74, 607.84, 607.82, 607.89, 607.9x; ICD-9 Pr: 64.94, 64.95, 64.96, 64.97; CPT: 54115, 54400-54402, 54405-54411, 54415, 54416, 54417, 54230, 54231, 54240, 54250, 54235, 37788, 37790 |
| Peyronie’s disease | All males | ICD-9 Dx: 607.81; ICD-9 Pr: 64.4 , 64.42; CPT: 54110, 54111, 54112, 54200, 54205, 54360 |
| Infertility | All males | ICD-9 Dx: 606.x, 606.0x, 606.1x, 606.8x, 606,9x, 456.4x; CPT: 54500, 54505, 54900, 54901, 55200, 55300, 55400, 55530, 55550, 55535, 55540, 55870, 52347, 74440 |
| Undescended Testis | All males | ICD-9 Dx: 752.5, 752.51, 752.52; ICD-9 Pr: 62.5x, 63.53; CPT: 54550, 54560, 54640, 54650, 54690, 54692, 54699 |
| Hypospadias | All males | ICD-9 Dx: 752.6 , 752.61; ICD-9 Pr: 58.45; CPT: 53430, 53450, 52270, 52283, 52276, 52282, 53400, 53405, 53410, 53415, 53420, 53425, 53431, 53450, 53000, 53010, 52283, 52275, 52276, 52282, 53020, 53025, 53600, 53601, 53602, 53605, 53620, 53621, 53640, 52281 |
| Urethral Stricture | All | ICD-9 Dx: 598.x, 598.1x, 598.2x, 598.8x, 598.9x, 598.0, 598.01; CPT: 53430, 53450, 52270, 52283, 52276, 52282, 53400, 53405, 53410, 53415, 53420, 53425, 53431, 53450, 53000, 53010, 52283, 52275, 52276, 52282, 53020, 53025, 53600, 53601, 53602, 53605, 53620, 53621, 53640, 52281 |
| Interstitial Cystitis | All | ICD-9 Dx: 595.1x, 595.2x, 595.9x, 596.9x, 625.8x, 625.9x, 788.41 |
| Prostatitis | All males | ICD-9 Dx: 601.0x, 601.2x, 601.3x, 601.4x, 601.8x, 601.1x, 601.9x |
| Prostate Cancer | Males 40 or older | ICD-9 Dx: 185.x, 233.4x, 236.5x; ICD-9 Pr: 60.5x, 60.62, 60.13, 62.4 , 62.42; CPT: 55810, 55812, 55815, 55840, 55842, 55845, 55859, 55860, 55862, 55865, 55873, 55866, J9217, J9218, J9219, J9202 |
| Bladder Cancer | All 40 or older | ICD-9 Dx: 189.1x, 189.2x, 189.3x, 188.xx, 233.7x; ICD-9 Pr: 56.5x, 57.6x, 57.4 , 57.49, 57.5 , 57.59, 57.71, 57.87, 58.39, 56.5x, 56.6x, 56.7x; CPT: 50234, 50236, 50548, 51020, 51530, 51570, 51575, 51580, 51585, 51590, 51595, 51596, 52224, 52234, 52235, 52240, 52250, 51720 |
| Kidney Cancer | All | ICD-9 Dx: 189.x, 189.0x, 189.8x; CPT: 502.30, 502.40, 505.43, 505.45 |
| Renal Mass | All | ICD-9 Dx: 593.9x, 239.5x |
| Testis Cancer | All males | ICD-9 Dx: 186.x, 186.0x, 186.9x; CPT: 54530, 54535, 38564, 38780 |

* Dx indicates diagnostic code; Pr, procedure code.
